# Rho-Kinase Inhibitor—A Molecule for Pharmacological Treatment of Decompensated Corneas: Case Series

**DOI:** 10.3390/biomedicines14051099

**Published:** 2026-05-13

**Authors:** Nina Kobal Mikša, Spela Stunf Pukl

**Affiliations:** 1 Faculty of Medicine, University of Ljubljana, 1000 Ljubljana, Slovenia; nina.kobal.nk@gmail.com; 2 Eye Hospital, University Medical Centre Ljubljana, 1000 Ljubljana, Slovenia

**Keywords:** Rho-kinase inhibitor, ROCK pathway inhibition, netarsudil, corneal endothelial dysfunction, corneal edema, corneal decompensation, corneal endothelial regeneration, endothelial barrier function, cytoskeletal modulation

## Abstract

**Objective:** Rho-associated protein kinase (ROCK) inhibitors have recently emerged as promising agents for the treatment of corneal endothelial dysfunction. Because corneal transparency critically depends on endothelial cell function, endothelial failure can lead to persistent visual impairment. However, clinical evidence regarding the use of topical ROCK inhibition in various etiologies of endothelial decompensation remains limited. The aim of this study was to evaluate changes in central corneal thickness (CCT), best-corrected visual acuity (BCVA), and treatment-related adverse events in eyes with corneal edema of different etiologies treated with fixed-combination drops of netarsudil 0.02%/latanoprost 0.005%, Roclanda^®^. **Methods:** In this prospective, uncontrolled, exploratory case series, we investigated the effects of topical ROCK inhibition on corneal endothelial cell function in 13 eyes of 11 patients with persistent, nonhealing corneal edema following intraocular procedures. Patients were treated with topical Roclanda^®^ once daily for three months. Clinical evaluation included BCVA, CCT, and safety assessment. Changes in CCT and BCVA were assessed before therapy, and after 1 and 3 months of treatment. **Results:** Mean baseline CCT was 782.8 µm and decreased significantly by 71.0 µm at 1 month and by 120.2 µm at 3 months (*p* = 0.0074 and 0.0012, respectively). Complete resolution of corneal edema was achieved in 38% of eyes. Mean BCVA improved from 0.744 before treatment to 0.518 logMAR at 3 months (*p* = 0.0026), with 46.2% of eyes gaining two or more Snellen lines. The analysis including only one eye per patient showed similar results, with statistically significant reductions in CCT at both 1 and 3 months and a significant improvement in BCVA at 3 months after the exclusion of the second eye in bilaterally included patients. Treatment was well tolerated; with mild conjunctival hyperemia as the most common adverse effect, while reticular epithelial corneal edema occurred in one eye and resolved after the completion of the treatment. **Conclusions:** In this prospective, exploratory case series of patients with nonhealing corneal edema, 3 months of a fixed-dose netarsudil 0.02%/latanoprost 0.005% treatment resulted in significant reduction in CCT, as well as clinically important improvement in BCVA. These exploratory findings cannot explain the mechanism of action, but suggest a potential therapeutic role for ROCK inhibitors in eyes with nonhealing corneal edema and possibly residual endothelial reserve. Larger controlled studies are needed to confirm these observations and further define indications for treatment.

## 1. Introduction

Corneal transparency is essential for optimal vision and depends on both, the highly organized structure of the cornea and the precise regulation of its dehydration. A key role in maintaining this balance is played by the corneal endothelium, which preserves stromal deturgescence through its pump–leak mechanism. While the hydrophilic glycosaminoglycans of the stromal extracellular matrix continuously attract fluid from the aqueous humor, endothelial cells actively remove ions and water from the stroma to counterbalance this influx. This tightly regulated equilibrium is crucial, as even small increases in corneal hydration beyond the normal physiological level of approximately 78% can impair transparency [1,2].

Endothelial cell loss or dysfunction, as they lack the proliferative capacity in vivo, represent the most common indication for corneal transplantation [3,4]. Endothelial cell deterioration can result from different etiologies, such as dystrophies (e.g., Fuchs endothelial corneal dystrophy, FECD; posterior polymorphous dystrophy), iatrogenic surgical trauma (e.g., pseudophakic bullous keratopathy following phacoemulsification) or toxic anterior segment syndrome after surgery, pathological conditions (e.g., iridocorneal endothelial syndrome, ICE; uveitis), and ocular trauma (e.g., contusion). These conditions lead to loss of corneal transparency due to reduction in viable endothelial cells, resulting in decreased number of Na^+^/K^+^-ATPase pumps on the cell membranes and, consequently, impaired endothelial pump function. This dysfunction causes stromal swelling, which initially develops in the posterior stromal layers and progressively advances to full-thickness stromal edema, and finally also microcystic epithelial edema. In advanced stages, when endothelial decompensation leads to persistent corneal edema and loss of transparency, corneal transplantation often remains necessary to restore visual function [5,6,7].

Pharmacologically induced regeneration of corneal endothelial cells has been proposed as a non-invasive approach to restoring endothelial pump function. As a topical therapy, it could significantly reduce the need for corneal transplantation, enable relatively rapid and effective visual recovery, and thereby substantially contribute to patients’ quality of life.

The Rho/Rho-associated protein kinase (ROCK) pathway is a key regulator of cellular behavior, particularly cytoskeletal dynamics, contractility, adhesion, and motility [8]. In ophthalmology, topical ROCK inhibitors were initially developed and approved for intraocular pressure reduction through effects on the trabecular meshwork and aqueous humor outflow. Netarsudil is approved for ocular hypertension and glaucoma and is also available as a fixed-dose combination with latanoprost, marketed as Rocklatan^®^ in the United States and Roclanda^®^ in Europe, while ripasudil has been approved for similar indications in Japan [9,10,11,12]. Beyond their pressure-lowering effect, experimental and early clinical studies suggest that ROCK inhibitors may support endothelial repair, barrier integrity, and pump function, thereby facilitating corneal deturgescence in selected eyes with residual endothelial reserve [13,14,15,16,17,18,19,20,21,22,23].

However, the clinical relevance of these mechanisms remains incompletely established, particularly in heterogeneous forms of persistent corneal edema and when using a fixed-dose netarsudil/latanoprost combination rather than netarsudil monotherapy. Therefore, the aim of this study was to evaluate changes in central corneal thickness (CCT), best-corrected visual acuity (BCVA), and treatment-related adverse effects in eyes with corneal edema of different etiologies treated with the fixed-dose netarsudil/latanoprost combination, which represents the only commercially accessible eye drops containing ROCK inhibitor in our region.

## 2. Materials and Methods

After the approval of the clinical study by the Commission of the Republic of Slovenia for Medical Ethics (approval no. 0120-580/2024-2711-4), a cohort of patients treated at the University Medical Centre Ljubljana for corneal decompensation with Roclanda^®^ was included into a prospective, uncontrolled, exploratory case series analysis. Participants were recruited between August 2024 and March 2025. All participants completed an informed consent process.

### 2.1. Inclusion and Exclusion Criteria

The study inclusion criteria were patients of any sex, who were at least 18 years of age, diagnosed with corneal edema after intraocular procedure, and able and willing to administer the study medication.

The exclusion criteria were active intraocular inflammation, corneal ulceration, keratitis, known sensitivity to any of the ingredients in the study medication; abnormal eyelid function; history of herpetic keratitis; history of noncompliance with using prescribed medication; current or planned pregnancy within the study duration, and any ocular or systemic condition that, in the investigator’s opinion, might put the patient at significant risk.

### 2.2. Study Protocol and Treatment Regimen

Before treatment initiation, all patients underwent a standardized ophthalmic assessment. Medical and ophthalmic history were recorded, including systemic and ocular comorbidities, previous ocular surgery, and concomitant medications. Corneal symptoms were assessed using the V-FUCHS questionnaire or an equivalent structured symptom questionnaire. Clinical examination included uncorrected distance visual acuity, BCVA using Snellen charts, intraocular pressure measurement, and slit lamp biomicroscopy.

Imaging assessments included corneal topography/tomography (Sirius+, CSO, Florence, Italy), anterior segment optical coherence tomography (AS-OCT; Anterion, Heidelberg Engineering, Heidelberg, Germany), and macular optical coherence tomography (Topcon, Tokyo, Japan). When corneal clarity allowed reliable image acquisition, specular microscopy (EM-3000, Tomey GmbH, Erlangen, Germany) was performed.

Patients were treated with topical fixed-dose netarsudil 0.02%/latanoprost 0.005% ophthalmic solution (Roclanda^®^, Santen Oy, Tampere, Finland), administered once daily in the evening for 3 months.

Follow-up examinations were performed at 1 week, 1 month, and 3 months after treatment initiation. The 1-week visit focused primarily on early safety assessment. At the 1- and 3-month visits, the clinical and imaging assessments performed at baseline were repeated. Treatment-related adverse effects were recorded at each follow-up visit.

### 2.3. Outcome Measures

The prespecified primary outcomes were the change in CCT from baseline to 1 month and from baseline to 3 months after treatment initiation. Secondary outcomes included the change in BCVA from baseline to 3 months and the occurrence of treatment-related adverse effects.

### 2.4. Statistical Analysis

CCT and BCVA values were summarized using descriptive statistics (mean and range). For statistical analysis, decimal Snellen visual acuity measurements were converted to logMAR values. The differences between baseline and follow-up measurements were assessed using the paired *t*-test, with statistical significance set at *p* < 0.05. Statistical analysis was performed using IBM SPSS Statistics for Windows, Version 24.0 (IBM Corp., Armonk, NY, USA).

## 3. Results

### 3.1. Clinical Cohort

A total of 13 eyes of 11 patients were included in the clinical cohort. Mean age was 78.7 years (range 65–90), and there were four male patients (five eyes).

A total of 11 corneas were decompensated after complicated cataract surgery (in one case a combined cataract surgery and vitrectomy for epiretinal membrane was done), in one eye after acute angular glaucoma attack and in one eye cornea did not clear up after Descemet stripping only due to FECD. Four eyes (three after cataract surgery and one after laser peripheral iridotomy) had decompensation in the form of bullous keratopathy. Four patients were also diagnosed with FECD (Table 1).

### 3.2. Outcomes

**Figure 1:**
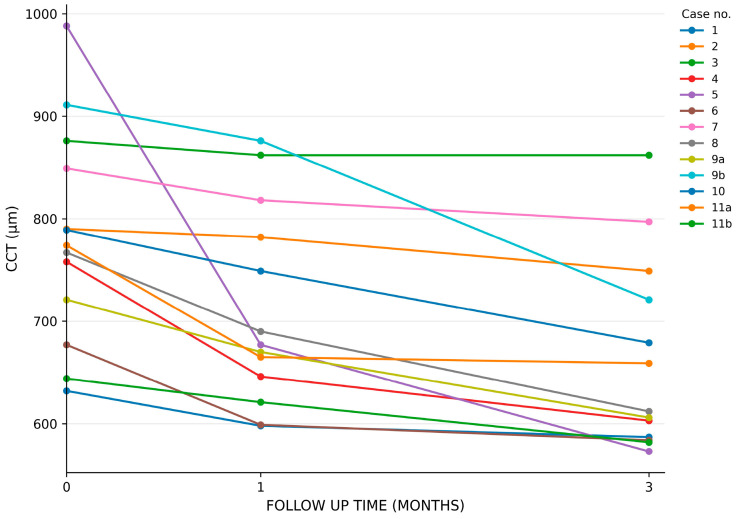
Spaghetti plot showing each eye’s central corneal thickness over time. Abbreviations: CCT—central corneal thickness.

**Figure 2:**
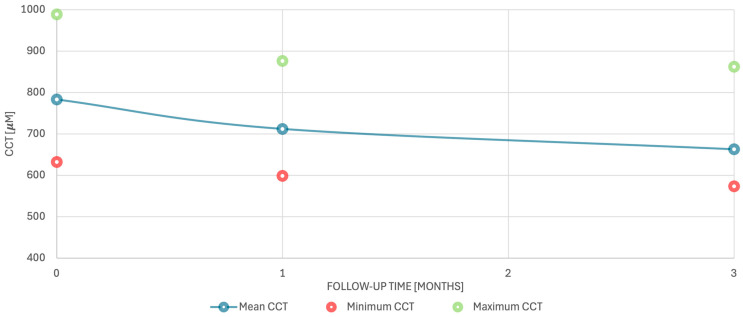
Central corneal thickness over time. Abbreviations: CCT—central corneal thickness.

The use of a fixed combination of netarsudil 0.02%/latanoprost 0.005% was associated with a significant decrease (improvement) in CCT between baseline and 1 month and between baseline and 3 months of treatment (Figure 1 and Figure 2). Baseline CCT (CCT0) was 783 µm (range 632–988 µm) (Table 2) (Figure 2). After 1 month of treatment, the mean reduction in CCT was 71 µm (range 8–311 µm), which was statistically significant (paired *t*-test, *p* = 0.0074). After 3 months of treatment, the mean reduction in CCT increased to 120 µm (range 14–415 µm), and this decrease was also statistically significant (paired *t*-test, *p* = 0.0012) (Table 2) (Figure 2). Because two patients (patient no. 9 and 11) contributed both eyes, the assumption of independence between eyes may have been partially violated. Inter-eye correlation was not formally modeled due to the small sample size. Therefore, the statistical findings should be interpreted cautiously. As a sensitivity analysis, all analyses were repeated including only one eye per patient. We retained the first treated eye (eye 9b and 11a) as this eye had more pronounced corneal decompensation and was associated with greater subjective symptoms. The fellow eye, which had milder symptoms and a lower degree of decompensation, was subsequently treated at the patient’s request after a favorable perceived response in the first treated eye. The analysis including only one eye per patient showed similar results. Mean baseline CCT was 801 µm (range, 632–988 µm), decreasing to 724 µm (range, 598–876 µm) at 1 month and 675 µm (range, 573–862 µm) at 3 months. The mean reduction in CCT remained statistically significant at both 1 month (77 µm; range 8–311 µm; *p* = 0.0134) and 3 months (126 µm; range 14–415 µm; *p* = 0.0037) (Table 3).

Complete resolution of corneal edema was documented in five eyes (38%). In the patient with corneal edema following an acute glaucoma attack, the treatment achieved sufficient corneal clearing to permit safe cataract surgery. In two additional eyes, edema decreased sufficiently to allow safe secondary IOL implantation. In four eyes, despite improvement in corneal edema, advanced stromal changes precluded satisfactory visual rehabilitation, and endothelial keratoplasty was therefore recommended.

At baseline, mean BCVA was 0.744 logMAR (range 0.155–2.301), improving to 0.518 logMAR (range 0.046–2.000) after 3 months of treatment (Table 4 and Table 5). This improvement was statistically significant (paired *t*-test, *p* = 0.0026), with a mean gain of 0.226 logMAR (range 0.000–0.778) (Table 4 and Table 5). The analysis including one eye per patient showed similar visual outcomes. Mean baseline BCVA was 0.795 logMAR (range, 0.155–2.301) and improved to 0.565 logMAR (range, 0.046–2.000) after 3 months of treatment. The mean improvement in BCVA was 0.230 logMAR (range, 0.000–0.778), which remained statistically significant (paired *t*-test, *p* = 0.0088) (Table 6). The improvement of two or more Snellen lines was documented in six of 13 eyes (46%). There were two cases where the corneal edema resolved completely, but had retinal changes limiting improvement in the visual outcome. In Case 1, despite complete edema resolution, visual acuity remained unchanged, likely due to concurrent macular changes—stage 4 epiretinal membrane; nevertheless, the patient reported subjective visual improvement. In Case 2, the treated eye had a history of branch retinal artery occlusion with optic nerve atrophy.

### 3.3. Safety Outcomes

The most commonly observed adverse effect was mild conjunctival hyperemia, which occurred in eight of 13 eyes (62%). Reticular epithelial edema developed in one eye. It was detected during planned follow-up visit. However, as the patient reported no associated symptoms or further deterioration in vision, the treatment was continued for three months, as planned. The edema resolved after treatment completion. Five patients reported irritation following instillation, while one patient described mildly increased photosensitivity. None of the adverse effects were considered sufficiently bothersome to warrant the discontinuation of the treatment. A summary of adverse effects is presented in Table 7.

## 4. Discussion

Corneal endothelial decompensation is a clinically significant cause of persistent corneal edema and visual impairment, as endothelial failure disrupts stromal deturgescence and reduces corneal transparency. In advanced or irreversible cases, endothelial keratoplasty remains the main treatment option. However, the limited availability of donor corneal tissue, together with the need to optimize or delay surgical intervention in selected patients, has increased interest in pharmacological approaches aimed at preserving or enhancing residual endothelial function. In this context, ROCK inhibitors have gained attention as potential therapeutic agents because of their ability to modulate endothelial cell behavior and possibly improve corneal barrier and pump function [1,2,3,4].

The ROCK pathway is a key regulator of cellular biomechanics and behavior, including cytoskeletal organization, contractility, adhesion, stiffness, and motility [8]. Its two major isoforms, ROCK1 and ROCK2, act as downstream effectors of the small GTPase RhoA and mediate actin cytoskeletal remodeling and actomyosin-driven contractile responses. Netarsudil directly inhibits both ROCK isoforms and interferes with phosphorylation of cytoskeletal target proteins, including myosin light chain, thereby promoting cytoskeletal relaxation. In the anterior segment, this mechanism contributes to increased aqueous humor outflow through the trabecular meshwork and Schlemm’s canal [9,10,11]. In addition, netarsudil inhibits the norepinephrine transporter, which may further contribute to intraocular pressure reduction by decreasing aqueous humor production and potentially lowering episcleral venous pressure [10]. Netarsudil is approved for the treatment of ocular hypertension and glaucoma and is also available as a fixed-dose combination with latanoprost, marketed as Rocklatan^®^ (Alcon, Geneva, Switzerland) in the United States and Roclanda^®^ (Santen Oy, Tampere, Finland) in Europe. Ripasudil, another topical ROCK inhibitor, has been approved in Japan for similar indications since 2014 [12].

Beyond intraocular pressure reduction, experimental studies have suggested that ROCK inhibition may have beneficial effects on the corneal endothelium. In vitro and ex vivo models have shown that ROCK inhibitors can promote corneal endothelial cell proliferation and migration, enhance intercellular adhesion, support endothelial wound healing, and inhibit apoptosis [13,14,15,16,17,18,19,20]. ROCK inhibition has also been associated with increased expression of genes and proteins involved in cell cycle regulation and endothelial pump function [19,21]. In animal models of experimentally induced endothelial injury, most commonly created by cryoinjury or Descemet membrane stripping, topical ROCK inhibitor treatment has been associated with improved corneal transparency, smaller residual wound areas, higher final endothelial cell density, reduced corneal edema, and better normalization of endothelial morphology compared with controls [13,14,17,22,23].

In the present prospective, uncontrolled, exploratory case series, the treatment with the fixed-dose netarsudil 0.02%/latanoprost 0.005% combination was associated with a significant reduction in CCT and improvement in BCVA from baseline to 3 months in selected eyes with corneal edema due to endothelial decompensation after intraocular procedures involving the anterior chamber. Given the small sample size, heterogeneous etiologies, and absence of a control group, these findings should be interpreted as exploratory and hypothesis-generating rather than as definitive evidence of efficacy.

These observations are broadly consistent with previous reports suggesting a potential role of ROCK inhibition in corneal endothelial dysfunction. However, direct comparisons with prior studies should be made cautiously, because available clinical evidence differs from the present cohort in disease etiology, baseline severity, chronicity of edema, treatment formulation, timing of intervention, follow-up duration, and study design.

Postoperative studies have primarily evaluated prophylactic or early postoperative ROCK inhibitor treatment. In the largest postoperative study, ripasudil 0.4% administered twice daily after cataract surgery was associated with significantly lower endothelial cell loss and a smaller increase in CCT compared with untreated controls [24]. Similarly, in a small series of three patients, perioperative ROCK inhibitor therapy was associated with less endothelial cell density loss than in the contralateral untreated eye [25]. In contrast, the present cohort included eyes with already established corneal edema and was treated with a fixed-dose netarsudil/latanoprost combination rather than ripasudil or netarsudil monotherapy.

Clinical studies on FECD provide additional relevant but not directly comparable evidence, as FECD differs from postoperative or secondary endothelial decompensation in natural history, endothelial reserve, and potential response to treatment. In the phase II of a randomized trial by Lindstrom et al., netarsudil was associated with a modest but measurable reduction in CCT, limited complete resolution of edema, and mild improvement in visual acuity, with once-daily dosing appearing at least as effective as twice-daily administration and possibly better tolerated [26]. Similarly, in a pilot placebo-controlled study, netarsudil treatment was associated with reduced CCT and improved visual acuity over three months [27]. Although the anatomical and functional changes observed in our cohort were numerically greater than those reported in some FECD studies, these differences should not be overinterpreted given the heterogeneity between studies. Therefore, our findings should be viewed as complementary exploratory observations supporting further investigation of ROCK inhibition in selected forms of endothelial decompensation, rather than as evidence of broad applicability across different causes of corneal edema.

In some eyes, treatment was associated with clinically meaningful corneal clearing. In the eye with corneal edema after an acute glaucoma attack, improvement in corneal clarity allowed subsequent cataract surgery. In two additional eyes, edema decreased sufficiently to permit secondary IOL implantation. These cases suggest that pharmacological treatment may have a potential role as a temporizing or bridging approach in selected eyes with residual endothelial reserve, improving corneal clarity sufficiently to allow secondary procedures to be performed more safely. However, this interpretation remains preliminary, and the present study was not designed to determine whether the treatment can reliably delay, replace, or reduce the need for endothelial keratoplasty.

At the same time, anatomical improvement did not always translate into proportional visual recovery. In one case, BCVA remained unchanged despite complete resolution of edema, most likely because of a stage 4 epiretinal membrane, although the patient reported subjective visual improvement. In another eye, complete edema resolution was achieved, but visual outcome was likely limited by previous branch retinal artery occlusion and optic nerve atrophy. Likewise, in four eyes, reduction in edema was not sufficient to allow meaningful visual rehabilitation because structural corneal changes were already too advanced, and endothelial keratoplasty was therefore offered. These observations underscore the importance of careful patient selection and suggest that the potential benefit of this treatment may depend on residual endothelial reserve, chronicity of edema, and the presence of irreversible corneal or posterior segment comorbidities.

A possible explanation for the observed reduction in corneal edema is that ROCK inhibition may improve endothelial barrier and pump function by modulating cytoskeletal tension and intercellular junction stability. Because endothelial junctional proteins are functionally linked to the actin cytoskeleton, the activation of the Rho/ROCK pathway may increase actomyosin-mediated contractility and junctional stress, thereby promoting paracellular leakage. Experimental studies suggest that ROCK inhibition can counteract these effects by stabilizing cell–cell junctions and decreasing endothelial permeability [28,29]. This concept is further supported by the in vitro study of Schlötzer-Schrehardt et al., in which netarsudil-induced cytoskeletal reorganization, reinforced tight junctions, reduced paracellular permeability, and upregulated proteins which are involved in endothelial pump function, including Na^+^/K^+^-ATPase, bicarbonate transporters, and aquaporins [21]. These changes were accompanied by enhanced proliferative and migratory capacity [21].

In the present clinical series, however, these mechanisms were not directly demonstrated. The observed reduction in CCT and improvement in BCVA may be consistent with improved endothelial barrier and/or pump function, but endothelial cell recovery, pump activity, and barrier integrity were not directly measured. Moreover, because all patients received the fixed-dose netarsudil/latanoprost combination, the observed clinical changes cannot be attributed to netarsudil alone. Although the ROCK-inhibitory component provides a biologically plausible rationale for edema reduction, a contribution of latanoprost cannot be excluded. Previous clinical studies have generally not shown a significant effect of latanoprost on endothelial cell density or endothelial morphology, although slight reductions in CCT have been reported [30,31,32]. Thus, rather than demonstrating a definitive mechanism, our findings support the hypothesis that treatment with the fixed-dose netarsudil/latanoprost combination may improve corneal hydration in selected eyes with residual endothelial reserve. This hypothesis remains biologically plausible but requires confirmation in larger controlled studies including standardized endothelial cell density, endothelial morphology, and longer-term follow-up data.

Treatment with the fixed-dose netarsudil/latanoprost combination was generally well tolerated in this cohort, with most adverse effects being mild and ocular-surface-related. The most frequently observed adverse effect was conjunctival hyperemia, followed by transient irritation after instillation and mild photosensitivity, which appeared in one patient. None of these were considered severe enough to require treatment cessation. Because all patients received the fixed-dose combination rather than netarsudil monotherapy, adverse effects cannot be attributed exclusively to netarsudil. Conjunctival hyperemia is a well-recognized adverse effect of ROCK inhibitors and is thought to be related to conjunctival vasodilation mediated by ROCK inhibition and altered vascular tone regulation [12,33]. However, conjunctival hyperemia and ocular surface symptoms are also known adverse effects of prostaglandin analogs, including latanoprost. Therefore, in the present cohort, hyperemia, irritation, and photosensitivity may reflect the effects of netarsudil, latanoprost, preservative-related ocular surface irritation, or a combination of these factors.

Reticular epithelial edema appears to be a rare but characteristic adverse effect reported during netarsudil therapy, particularly in eyes with pre-existing endothelial dysfunction [12]. In our cohort, this finding was observed in one patient and resolved after the completion of the planned treatment, consistent with the reversible course described in previous reports [34]. Importantly, this patient had pre-existing epithelial bullous changes in the setting of bullous keratopathy before the initiation of therapy, which may have increased susceptibility to this complication. Clinically, netarsudil-associated reticular epithelial edema is characterized by superficial microcystic epithelial bullae arranged in an interconnected honeycomb-like pattern, typically confined to the epithelium [34]. Reported cases suggest that affected eyes often have substantial underlying endothelial compromise or related risk factors, including prior corneal transplantation, FECD, glaucoma drainage devices, chronic glaucoma with elevated intraocular pressure, inflammatory endothelial damage, or pre-existing epithelial bullous changes [35,36,37,38].

At the cellular level, this phenomenon may reflect differential effects of ROCK inhibition on the corneal endothelium and epithelium. Whereas the endothelial effects of netarsudil have been discussed above, its effects on epithelial barrier integrity may help explain the development of reticular epithelial edema [21]. In epithelial cells, ROCK inhibition has been shown experimentally to destabilize cell–cell and cell–matrix adhesion, disrupt tight junction architecture, alter localization and expression of junctional proteins such as ZO-1, occludin, and claudin-1, and reduce the expression of hemidesmosomal components including integrin α6 and β4, leading to increased paracellular permeability and reduced transepithelial electrical resistance [21,39]. These epithelial changes appear to be reversible after drug withdrawal, which is consistent with the clinical resolution of reticular epithelial edema observed in our patient and in previously published cases [34]. Careful slit lamp monitoring is therefore advisable in eyes with bullous keratopathy, epithelial bullae, severe endothelial decompensation, previous corneal transplantation, or other risk factors for epithelial barrier instability.

The main limitations of this study are its small sample size, uncontrolled design, heterogeneous etiologies of corneal edema, and absence of a comparator group. These factors limit causal inference and make it difficult to exclude spontaneous improvement, regression toward the mean, or differences in the natural course of the underlying conditions. Possible inter-eye correlation was further taken into account by a sensitivity analysis including only one eye per patient, and similar and statistically significant results were proved. Another shortcoming was the inability to obtain specular microscopy of the endothelium through opacified stroma, which would demonstrate the density and morphological cell changes. Therefore, the present findings should be interpreted as exploratory clinical observation, and larger controlled studies with morphological confirmation are needed to confirm the definitive evidence of efficacy, mechanism of action, and clinical applicability.

## 5. Conclusions

In this prospective, uncontrolled, exploratory case series, treatment with the fixed-dose netarsudil 0.02%/latanoprost 0.005% combination was associated with a significant reduction in CCT and improvement in BCVA in selected eyes with corneal edema due to endothelial decompensation after intraocular procedures. The observed reduction in corneal edema may be consistent with improved endothelial barrier and/or pump function in eyes with some residual endothelial reserve; however, this mechanism was not directly demonstrated in the present clinical series. Therefore, treatment with fixed-dose netarsudil/latanoprost may warrant further investigation as a potential adjunctive or temporizing pharmacological approach in selected eyes with corneal edema. Its use should be considered cautiously in eyes with severely reduced endothelial reserve, advanced structural corneal changes, or epithelial barrier disruption.

In selected cases, this treatment may have a potential role as a bridging therapy while awaiting endothelial keratoplasty or as a temporary strategy to improve corneal clarity and symptoms. However, definitive conclusions regarding efficacy, optimal patient selection, treatment duration, and broader clinical applicability cannot be drawn from this case series.

Larger controlled clinical studies with standardized endothelial assessment and longer follow-up are needed to confirm these observations, as well as to more precisely define which corneal changes will most likely respond to treatment and thus identify patients who would benefit from such treatment.

## Figures and Tables

**Table 1:** Cohort patients’ characteristics and central corneal thickness over time.

Case Number	Sex	Age (Years)	Etiology of Corneal Decompensation	Additional Diagnoses	Duration of Postoperative Edema (Days)	CCT0 (μm)	CCT1 (μm)	CCT3 (μm)
1	M	86	Edema after cataract surgery	Epiretinal membrane	284	632	598	587
2	F	87	Edema after cataract surgery	Status post branch retinal artery occlusion	445	790	782	749
3	M	89	Edema after complicated cataracy surgery (bullous keratopathy)		150	876	862	862
4	M	76	Edema after complicated cataract surgery	Postoperative aphakia, postoperative cystoid macular edema	186	758	646	603
5	F	82	Edema after complicated cataract surgery	Postoperative aphakia	48	988	677	573
6	F	75	Edema after complicated cataract surgery combined with vitrectomy	Epiretinal membrane	1046	677	599	584
7	F	81	Edema after Descemet stripping only	FECD	236	849	818	797
8	F	73	Edema after acute angle-closure attack (bullous keratopathy)		251	767	690	612
9a	M	73	Edema after cataract surgery	FECD	311	721	670	606
9b	M	73	Edema after cataract surgery (bullous keratopathy)	FECD	259	911	876	721
10	F	69	Edema after cataract surgery (bullous keratopathy)	FECD	128	789	749	679
11a	F	64	Edema after cataract surgery	FECD	62	774	665	659
11b	F	64	Edema after cataract surgery	FECD	110	644	621	582

Abbreviations: F—female; M—male; CCT0—baseline corneal thickness; CCT1—corneal thickness after 1 month of treatment; CCT3—corneal thickness after 3 months of treatment; FECD—Fuchs endothelial corneal dystrophy.

**Table 2:** Mean corneal thickness and range over time and mean reduction from baseline.

Mean CCT0 (µm)	Mean CCT1 and Mean Reduction (µm)	Mean CCT3 and Mean Reduction (µm)
783 (632–988)	712 (598–876)	663 (573–862)
	−71 (8–311)	−120 (14–415)

Abbreviations: CCT0—baseline corneal thickness; CCT1—corneal thickness after 1 month of treatment; CCT3—corneal thickness after 3 months of treatment.

**Table 3:** Mean corneal thickness and range over time and mean reduction from baseline when the second treated eye in bilateral patients was excluded.

Mean CCT0 (µm)	Mean CCT1 and Mean Reduction (µm)	Mean CCT3 and Mean Reduction (µm)
801 (632–988)	724 (598–876)	675 (573–862)
	−77 (8–311)	−126 (14–415)

Abbreviations: CCT0—baseline corneal thickness; CCT1—corneal thickness after 1 month of treatment; CCT3—corneal thickness after 3 months of treatment.

**Table 4:** Change in BCVA after 3 months of treatment.

Case Number	Baseline BCVA in Snellen Decimal	Baseline BCVA (logMAR)	BCVA After 3 Months of Treatment in Snellen Decimal	BCVA After 3 Months of Treatment in logMAR	Change in BCVA (Baseline—3 Months) in logMAR
1	0.2	0.699	0.2	0.699	0.0
2	0.16	0.796	0.3	0.523	0.273
3	0.005	2.301	0.01	2.00	0.301
4	0.2	0.699	0.3	0.523	0.176
5	0.1	1.0	0.6	0.222	0.778
6	0.16	0.796	0.3	0.523	0.273
7	0.7	0.155	0.9	0.046	0.109
8	0.2	0.699	0.2	0.699	0.0
9a	0.5	0.301	0.7	0.155	0.146
9b	0.3	0.523	0.5	0.301	0.222
10	0.1	1.0	0.3	0.523	0.477
11a	0.4	0.398	0.6	0.222	0.176
11b	0.5	0.301	0.5	0.301	0.0

Abbreviations: BCVA—best-corrected visual acuity.

**Table 5:** BCVA over time in logMAR.

	Mean	Minimum	Maximum
Baseline BCVA	0.744	0.155	2.301
BCVA after 3 months	0.518	0.046	2.00
Improvement in BCVA (baseline—3 months)	0.226	0.0	0.778

Abbreviations: BCVA—best-corrected visual acuity.

**Table 6:** BCVA over time in logMAR when the second treated eye in bilateral patient was excluded.

	Mean	Minimum	Maximum
Baseline BCVA	0.795	0.155	2.301
BCVA after 3 months	0.565	0.046	2.00
Improvement in BCVA (baseline—3 months)	0.230	0.0	0.778

Abbreviations: BCVA—best-corrected visual acuity.

**Table 7:** Adverse effects.

Adverse Effect	Number of Patients Affected	Severity	Time to Onset	Management	Outcome
Conjunctival hypermia	8/13 (62%)	Mild to moderate	In the first week of treatment	Treatment continued	Resolved after completion of planned treatment
Irritation after instillation	5/13 (38%)	Mild to moderate	Immediately	Treatment continued	Resolved in a few minutes
Increased photosensitivity	1/13 (8%)	Mild	In the first week of treatment	Treatment continued	Resolved after completion of planned treatment
Reticular epithelial edema	1/13 (8%)	Mild to moderate	In the second month of treatment	Treatment continued	Resolved after completion of planned treatment

## Data Availability

The data presented in this study are available on request from the corresponding author due to ethical restrictions related to patient confidentiality.

## Abbreviations

The following abbreviations are used in this manuscript:

AS-OCT	anterior segment optical coherence tomography
BCVA	best-corrected visual acuity
CCT	central corneal thickness
FECD	Fuchs endothelial corneal dystrophy
ICE	iridocorneal endothelial syndrome
OCT	optical coherence tomography
ROCK	Rho/Rho-associated protein kinase
U.S. FDA	United States Food and Drug Administration
